# Cardiac Dedifferentiated Liposarcoma Requiring a Mitral Valve Replacement Complicated by Severe Paravalvular Leak: A Rare Case Report with Literature Review

**DOI:** 10.1155/2018/2506368

**Published:** 2018-09-04

**Authors:** Anju Adhikari, Adam Hafeez, Alexandra Halalau

**Affiliations:** ^1^Internal Medicine Department, Beaumont Hospital, Royal Oak, MI, USA; ^2^Oakland University William Beaumont School of Medicine, Rochester, MI, USA

## Abstract

Cardiac sarcomas have a high infiltrative and metastatic potential and are often associated with poor prognosis. These tumors are often identified incidentally by thoracic and cardiac imaging. However, when symptomatic, their presentation can differ based on the localized structural effects on the endocardium, myocardium, pericardium, and valves as well as on dynamic effects on the cardiac function. We report a case of a 61-year-old female who presented to the emergency room with recurrent chest pain, fatigue, and chronic anemia. A transthoracic echocardiogram demonstrated a left atrial mass attached to the septal wall and anterior leaflet of the mitral valve. The mass was further characterized by a transesophageal echocardiogram and cardiac MRI. The patient underwent a resection of the left atrial mass with mitral valve replacement (MVR) and atrial septal defect repair. MVR was later complicated by paravalvular leak leading to acute congestive heart failure. Tissue immune histology was consistent with dedifferentiated liposarcoma. Cardiac dedifferentiated liposarcoma is extremely rare with only few cases reported in literature. We attempt to review the clinical features, diagnosis, and management of cardiac sarcoma with great emphasis.

## 1. Background

Cardiac tumors in general are relatively rare and can be either primary or secondary. Secondary cardiac involvement from metastasis and local extension are slightly more common, although still relatively rare when compared with other organ systems. Secondary metastatic tumors are commonly known to arise from the malignancies originating in the lungs, breast, kidney, skin, or lymphatic tissue (lymphoma) [1]. On the other hand, primary cardiac tumors are known to be extremely rare with some studies reporting less than 0.1 percent incidence [2].

Primary cardiac tumors are commonly benign while malignant primary cardiac tumors remain very rare with autopsy series reporting a prevalence of 0.001% [3]. The Japanese Association of Thoracic Surgery (JATS) along with the Japanese Circulation Society (JCS) compiled a compendium of cardiac tumors from 1999 to 2010 [4]. They found that the most common benign cardiac tumor was myxoma (34.0–43.1%), followed by papillary fibroelastoma (11.4–17.7%). This was followed by lipoma and hemangioma in this order. On the other hand, they found that malignant cardiac tumors were most commonly angiosarcoma (8.2–9.5%), followed by fibrous histiocytoma and leiomyosarcoma. Cardiac dedifferentiated liposarcoma is extremely rare with less than 10 cases reported in the literature. It is a type of liposarcoma with an infiltrative and metastatic potential. We report a case of a female presenting with cardiac liposarcoma of primary cardiac origin.

## 2. Case

**Figure 1:**
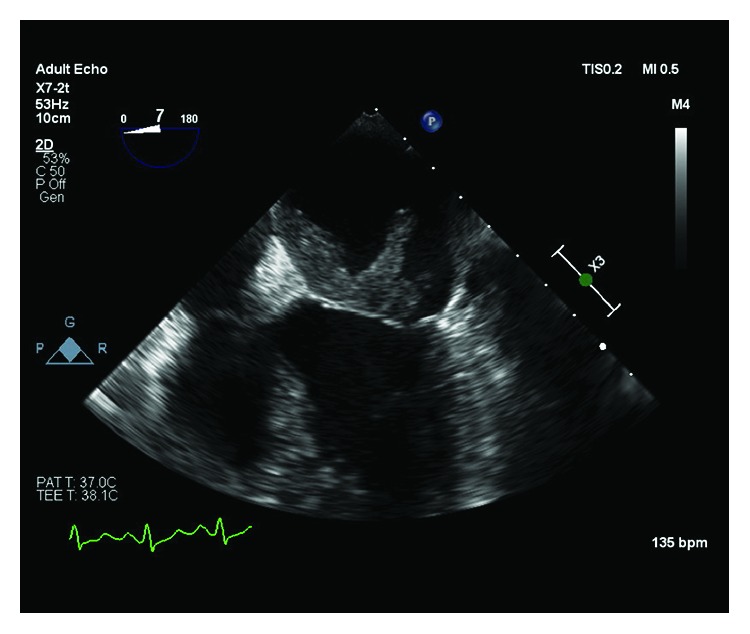
Preoperative midesophageal TEE 4-chamber view showing a large echo-dense atrial mass; one segment is attached to the atrial septum (2.67 cm × 1.43 cm) and another segment is attached to the anterior mitral leaflet (1.43 cm × 2.22 cm).

**Figure 2:**
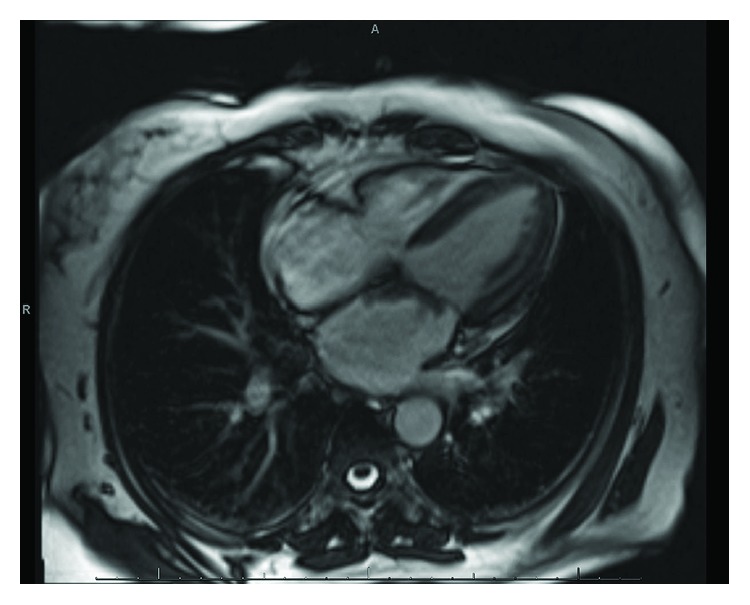
Cardiac MRI first-pass perfusion imaging showing mass originating on the atrial septum and extending along the atrial aspect of the anterior mitral valve leaflet.

A 61-year-old female with past medical history significant for type 2 diabetes mellitus, hypertension, and dyslipidemia and family history of breast cancer in her brother and maternal aunt presented to the emergency room for recurrent chest pain. She also reported fatigue for the past few months and was being evaluated for worsening anemia by her primary care physician. Her hemoglobin was 6 gm/dl with iron panel consistent with anemia of chronic disease. Her electrocardiogram (ECG) at arrival showed an “RSR pattern” in V1 and V2 leads but was otherwise normal. She underwent a stress myocardial perfusion imaging (MPI) which was negative for cardiac ischemia. A transthoracic echocardiogram revealed a 1.6 cm × 1.5 cm atrial mass attached to the anterior wall of the left atrium, which appeared to cross the mitral valve in ventricular diastole. The left atrial mass was further characterized by a transesophageal echocardiogram (TEE) as a solid, irregularly shaped, partly mobile mass attached to the atrial septum and extending to the anterior mitral valve leaflet (Figure 1). The segment attached to the septum measured 2.6 cm × 1.43 cm, and the segment attached to the valve measured 1.4 cm × 2.22 cm. In addition, MRI of the heart with gadolinium was done preoperatively which confirmed the circumscribed hypodense mass with speckled appearance which originated at the atrial septum and extended along the anterior mitral valve leaflet (Figure 2). The mitral valve flow was normal with no evidence of obstruction, stenosis, or regurgitation. The patient underwent a minimal incision valve surgery for resection of the mass which was presumed to be myxoma due to its location. Intraoperatively, on open examination of the left atrium, it was noted that the mass originated from the fossa ovalis region of the interatrial septum and infiltrated the atrial wall down onto the entire anterior leaflet of the mitral valve. A fibrotic density that surrounded the tumor was also noted. The mass along with a portion of the interatrial septum and the mitral valve was resected. The mitral valve was replaced using a 27 mm Hancock II bioprosthetic valve. The atrial septal defect caused by the resection was repaired with a bovine pericardial patch. A postoperative TEE was performed which confirmed the successful placement of the bioprosthetic valve with no paravalvular leak. The patient had an otherwise unremarkable postoperative recovery and was discharged home after fourteen days of hospital stay.

**Figure 3:**
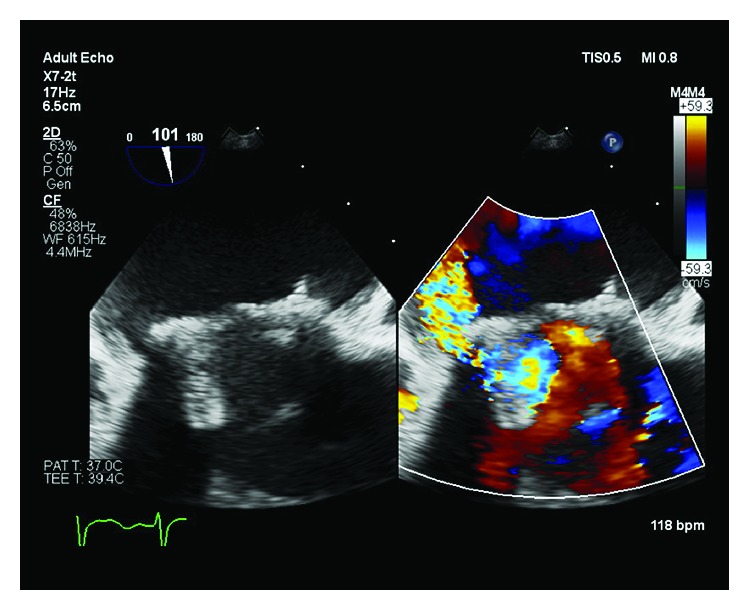
TEE 2-chamber view at hospital readmission showing severe paravalvular leak of the bioprosthetic mitral valve. 180° rotation showed leak extending along the medial aspect of the valve from the posterior to the anterior.

**Figure 4:**
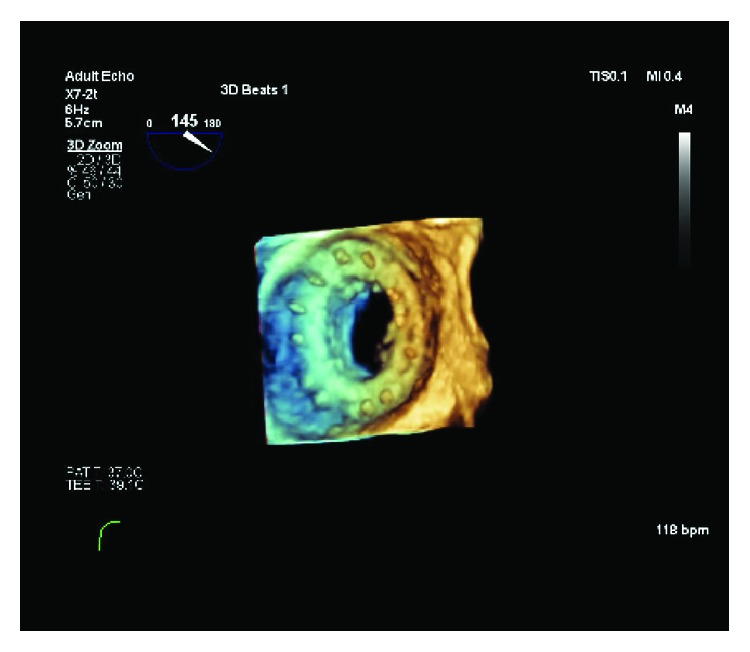
TEE 3D reconstruction showing bioprosthetic mitral valve in the short axis with paravalvular dehiscence that extends along the medial aspect of the valve from the posterior to the anterior.

**Figure 5:**
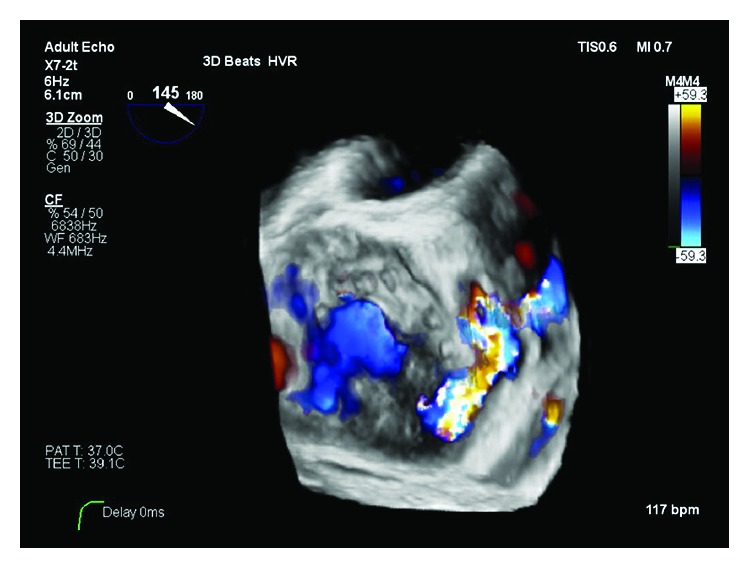
TEE 3D reconstruction with color Doppler showing a bioprosthetic mitral valve with paravalvular leak on the short axis.

**Figure 6:**
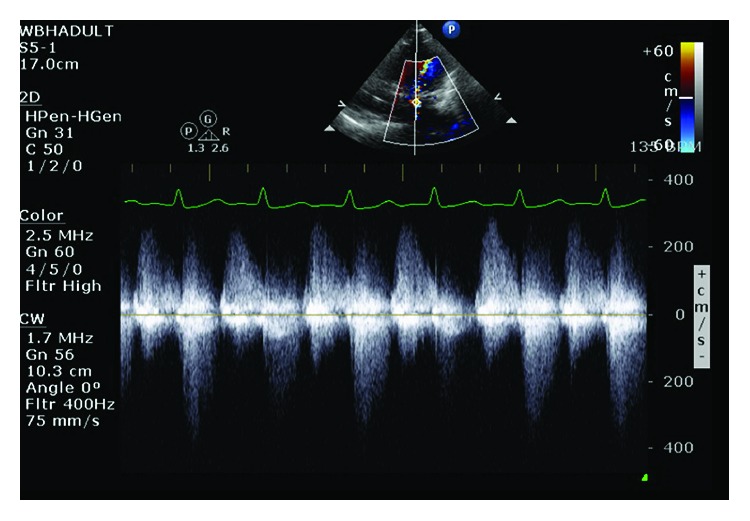
TEE continuous wave showing severe mitral regurgitation.

**Figure 7:**
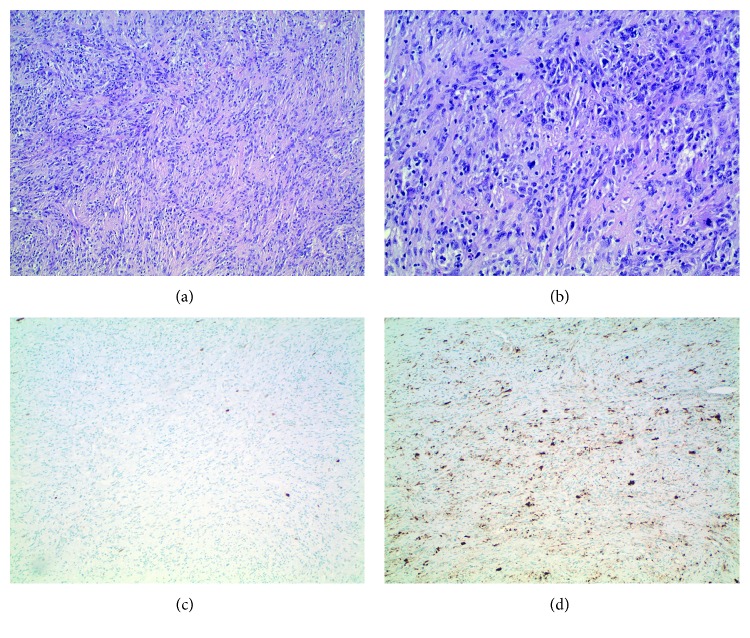
(a) 10x H&E, (b) 20x H&E, (c) tumor negative for p53, and (d) tumor focally positive for S100.

Two weeks following discharge, the patient presented to the emergency with cough, diaphoresis, and palpitations. She was noted to have jugular venous distention and bibasilar crackles on auscultation of the lungs and was found to be in acute heart failure. An urgent transesophageal echocardiography demonstrated severe mitral regurgitation with paravalvular leak (Figures 3, 4, 5, and 6). At the same time, the histopathologic examination of the atrial mass showed a high-grade sarcoma consistent with dedifferentiated liposarcoma. The tissue exhibited spindle cells with pleomorphism, multinucleated giant cells, and inflammatory cells. Immunohistochemical stains demonstrated that the neoplastic cells were positive for vimentin, focally positive for S-100, and weakly positive for CDK4 and negative for p53 (Figure 7). FISH studies performed showed an MDM-2 gene amplification in 95–200 nuclei examined. The patient was aggressively treated with intravenous diuretics and afterload reduction using furosemide and nicardipine infusion. Blood cultures were obtained with suspicion of postsurgical infective endocarditis causing valvular dehiscence. However, cultures did not grow any bacteria. The patient clinically deteriorated due to new-onset atrial fibrillation and worsening heart failure despite medical treatment in CCU. CT chest obtained showed a 1.3 cm lytic iliac bone lesion and 3.1 cm × 2.5 cm right upper mediastinal soft tissue density. Due to high suspicion of metastatic disease, MVR and cardiac transplant were not offered until further evaluation for metastasis. Unfortunately, due to rapid clinical decline with a new diagnosis of high-grade cardiac tumor with possible metastases, the patient opted for hospice care. PET study was not obtained.

## 3. Discussion

Among malignant cardiac tumors, cardiac sarcomas appear to be the most common. In a retrospective study done in Germany from 1989 to 2012 that looked at 181 diagnosed cardiac tumors, cardiac sarcoma represented 75% of all malignant cardiac tumors [5]. Histologically, soft tissue sarcomas in general have been classified by the World Health Organization (WHO) to include more than 100 different histologic subtypes on the basis of the morphologic pattern most often noted on immunohistochemical staging (IHC) [6]. The most common soft tissue sarcoma subtypes in adults include undifferentiated/unclassified soft tissue sarcoma, undifferentiated pleomorphic sarcoma, liposarcoma, leiomyosarcoma, and gastrointestinal stromal tumors (GIST). Liposarcoma constitutes only 13% of all cardiac sarcomas, arises from adipocytes, and has three main morphologic subgroups that vary in their metastatic potential according to 2013 WHO classification of soft tissue sarcoma: (1) myxoid, (2) dedifferentiated, and (3) pleomorphic liposarcoma [7]. Myxoid liposarcoma are low-grade tumors while dedifferentiated and pleomorphic liposarcomas are highly aggressive tumors. Dedifferentiated liposarcoma, as seen in our patient, is extremely rare with less than 10 reported cases in PubMed and Embase together (Table 1). However, dedifferentiated liposarcomas of the mediastinum, retroperitoneum, and extremities were more commonly reported.

Common clinical symptoms of cardiac tumors include dyspnea, orthopnea, paroxysmal nocturnal dyspnea, hemoptysis, edema, and nonspecific constitutional symptoms such as fevers, chills, or fatigue, arthralgias, and myalgias [8]. Laboratory studies are inconsistent and may show anemia due to chronic disease, mildly elevated white blood cell count, and elevated erythrocyte sedimentation rate. Tumors can cause direct obstruction, valvular dysfunction, coronary artery spasm, changes in the electrical conduction of the heart with arrhythmias, and production of a malignant pericardial effusion. Cardiac tumors may metastasize or embolize causing distant complications with brain infarction being a feared complication. One study on atrial myxomas reported as high as 12% incidence of central nervous system complications from left atrial tumors [9].

Left atrial tumors are commonly presumed to be benign myxomas. Although left atrial tumors are commonly benign, the possibility of malignancy should always be considered given that 50% of cardiac sarcomas occur in the left atrium [10]. As shown in Table 1, most reported cases of dedifferentiated liposarcoma arose in the left atrium. These tumors are known to impede blood flow and cause mitral valve obstruction. Clinically, patients often present with symptoms of heart failure or pulmonary hypertension (Table 1). Although not commonly reported, physical exam maneuvers may produce a tumor plop in early diastole.

Evaluation for cardiac tumors rests heavily on imaging modalities. There are three major imaging modalities that aid with the diagnosis of cardiac tumors. Transthoracic echocardiography remains at the heart of diagnosing and screening of cardiac neoplasms as it has a high sensitivity and specificity (90% and 95%, respectively) for detecting intracavitary and endocardial lesions [1, 11]. TEE can be utilized for a more accurate evaluation as it provides superior spatial resolution and is optimal for tumors where areas such as the atria, interatrial septum, superior vena cava, and atrioventricular valves are difficult to visualize. In our patient, TEE provided a more accurate view of the tumor size, extent, and attachment than the initial TTE. MRI is currently the modality of choice to evaluate tissue infiltration, pericardial image, and extracardiac extension whenever a malignant lesion is suspected. Multidetector CT is a relatively new modality which produces images with higher spatial resolution in a shorter exposure span and also better demonstrates calcifications, fatty lesions, and cardiac valves [12]. Clinical signs and symptoms along with imaging can help formulate a presumed diagnosis of a cardiac tumor but, as seen in our patient, cannot be relied on to help differentiate between benign or metastatic diseases. The tumor stage and grade can only be made with histological confirmation. Minimally invasive techniques can be used if the malignancy is in pericardial fluid or if an imaging-guided percutaneous biopsy can be performed. However, in endocardial tumors, the risk of embolization often outweighs the benefits of biopsy of the tumor, given that the tumor will eventually be resected anyways. Of note, percutaneous needle biopsy is not adequate for the diagnosis and grade determination of lipomatous tumors. These tumors require larger tissue specimens obtained by incisional or excisional biopsy which further complicates and delays their diagnosis [5].

Surgical resection with or without chemotherapy or radiotherapy remains the treatment of choice for cardiac malignancies including liposarcoma. Management begins by assessing the extent and size of tumor as that determines the approach to excise such tumors. Positron emission tomography (PET) as well as coronary angiography is important as mapping the blood supply of tumors helps cardiac surgeons know where to also resect or graft involved arteries. Simple tumor resection is selected for benign tumors with no infiltration while complex tumor resection and ex situ resection are utilized for malignant tumors. Implantation of an artificial heart and heart transplantation are reserved for young patients with no evidence of distant metastasis [5].

Unfortunately, sarcomas in general, regardless of the morphologic type, are known to rapidly proliferate. They are known to infiltrate the myocardium, obstruct blood flow through the heart, and cause metastasis. In a study of 181 patients with cardiac tumors, among which 25% were malignant, the 5-year survival rates for malignant tumors following treatment was 30% [5]. The study also reported that complete tumor resection and adjuvant radiation therapy contributed to the reduced risk of local recurrence and metastasis. Specifically with liposarcoma, recurrence was seen in approximately 40% of the cases and was reported to be up to 14 years after the initial surgical resection [7].

Our patient developed paraprosthetic leak following mitral valve replacement (MVR). Mild paravalvular leak (PVL) is a fairly common complication occurring in almost 20% post-MVR. Of note, however, clinically significant PVL occurs in only 1–5% [13]. Calcification and fibrosis of the annulus are known to increase the risk of PVL. On the other hand, it is well known that malignant tissue often displays fibrosis or calcification. Our patient had fibrotic changes around the atrial cancer which was easily noticed during resection. Hence, it is possible that the remnant friable malignant tissue or its sclerotic surrounding tissue might have contributed to the PVL in our patient leading to poor clinical outcome. Keeping in mind the limitations of echocardiogram, cardiac MRI, and CT in diagnosing a malignant tumor, we suggest the utilization of PET scan whenever malignancy is suspected. Presurgical diagnosis of malignant tumors would not only help surgeons plan a complete tumor resection and anticipate complications but would also allow neoadjuvant radiation or chemotherapy. At present, the prognosis of cardiac malignancy remains poor with high mortality and tumor recurrence. The advent of new modalities to diagnose cardiac malignancy before curative surgery could potentially decrease the mortality in the future.

## Figures and Tables

**Table 1:** Case reports of cardiac dedifferentiated liposarcoma indexed in PubMed and Embase.

Number	Publication	Year	Primary/Metastatic	Location of tumor	Presentation
1	Seaweed floating in the pericardium: a rare case of primary dedifferentiated liposarcoma [14]	2016	Primary	Pericardium	Dyspnea
2	Primary cardiac dedifferentiated liposarcoma with homologous and heterologous differentiation: a case report [15]	2015	Primary	Left atrium	Dyspnea on exertion, fatigue, palpitation
3	Large left ventricular metastasis in patient with liposarcoma [16]	2016	Metastasis from pleura	Left ventricle	Not mentioned
4	Synchronous primary heart liposarcoma and papillary renal carcinoma--a case report (abstract only) [17]	2003	Not available	Not available	Not available
5	Metastatic cardiac liposarcoma of the small intestine causing enteroenteric intussusception: a case report and a review of the literature [18]	2015	Primary with metastasis to the small intestine	Left atrium	Dyspnea on exertion
6	A case of dedifferentiated liposarcoma of the heart and stomach [19]	2017	Unknown	Left atrium	Anorexia and weight loss
7	Mitral stenosis secondary to high-grade liposarcoma in a young pregnant [20]	2015	Primary	Left atrium and right pulmonary vein	Acute dyspnea
8	Multiple skeletal muscle metastases revealing a cardiac intimal sarcoma [21]	2018	Primary cardiac with skeletal muscle metastasis	Left atrium	Thigh swelling
